# Usage of 3D models of tetralogy of Fallot for medical education: impact on learning congenital heart disease

**DOI:** 10.1186/s12909-017-0889-0

**Published:** 2017-03-11

**Authors:** Yue-Hin Loke, Ashraf S. Harahsheh, Axel Krieger, Laura J. Olivieri

**Affiliations:** 1 Division of Cardiology, Children’s National Health System, 111 Michigan Ave NW, Washington, DC 20010-2970 USA; 2 Bioengineering Institute, Sheikh Zayed Institute for Pediatric Surgical Innovation, Children’s National Health System, 111 Michigan Ave NW, Washington, DC 20010-2970 USA

**Keywords:** Congenital heart disease, 3D printing, Resident education

## Abstract

**Background:**

Congenital heart disease (CHD) is the most common human birth defect, and clinicians need to understand the anatomy to effectively care for patients with CHD. However, standard two-dimensional (2D) display methods do not adequately carry the critical spatial information to reflect CHD anatomy. Three-dimensional (3D) models may be useful in improving the understanding of CHD, without requiring a mastery of cardiac imaging. The study aimed to evaluate the impact of 3D models on how pediatric residents understand and learn about tetralogy of Fallot following a teaching session.

**Methods:**

Pediatric residents rotating through an inpatient Cardiology rotation were recruited. The sessions were randomized into using either conventional 2D drawings of tetralogy of Fallot or physical 3D models printed from 3D cardiac imaging data sets (cardiac MR, CT, and 3D echocardiogram). Knowledge acquisition was measured by comparing pre-session and post-session knowledge test scores. Learner satisfaction and self-efficacy ratings were measured with questionnaires filled out by the residents after the teaching sessions. Comparisons between the test scores, learner satisfaction and self-efficacy questionnaires for the two groups were assessed with paired *t*-test.

**Results:**

Thirty-five pediatric residents enrolled into the study, with no significant differences in background characteristics, including previous clinical exposure to tetralogy of Fallot. The 2D image group (*n* = 17) and 3D model group (*n* = 18) demonstrated similar knowledge acquisition in post-test scores. Residents who were taught with 3D models gave a higher composite learner satisfaction scores (*P* = 0.03). The 3D model group also had higher self-efficacy aggregate scores, but the difference was not statistically significant (*P* = 0.39).

**Conclusion:**

Physical 3D models enhance resident education around the topic of tetralogy of Fallot by improving learner satisfaction. Future studies should examine the impact of models on teaching CHD that are more complex and elaborate.

## Background

Congenital heart disease (CHD) is the most common human birth defect, with an estimated incidence of 8 per 1,000 live births [1]. Each defect has a wide spectrum of severity. The clinical impact and subsequent management of the CHD largely depends on anatomy. Given the need for pediatricians to direct and coordinate healthcare delivery for patients with CHD, a basic understanding of common CHD is necessary [2]. However, the subspecialization in pediatric cardiology limits the clinical exposure of pediatric residents to CHD [3], creating knowledge gaps in the clinical management of CHD [4]. Furthermore, the standard technique of visualizing CHD, such as echocardiography, cardiac catheterization, or cross-sectional CT/MRI, requires mentally reconstructing multiple planes of 2D images into a 3D object. This technique is difficult to learn, and often disorienting considering the heart’s complex anatomy.

Three-dimensional (3D) models may be useful in improving the understanding of CHD, without requiring a mastery of cardiac imaging. Previous studies in other medical disciplines have shown that digital 3D models can supplement traditional instructional methods, and are superior to 2D images in the teaching of complex anatomy (such as larynx [5] or the knee joint [6]). In Bigilio et al’s study, printed 3D models from children with recent cardiac MRIs were used during outpatient clinic discussions of CHD with family members [7]. Printed 3D models of ventricular septal defects have previously been utilized in ICU simulation-based instructions, to teach postoperative critical care management of ventricular septal defects [8, 9]. Experiences from such case studies suggest that utilizing physical CHD models help with physicians’ understanding of congenital heart disease.

The objective of this study is to evaluate the impact of 3D models on how pediatric residents understand tetralogy of Fallot during a teaching session. Tetralogy of Fallot was selected as the topic, as this common lesion is frequently encountered by general pediatricians. The dynamic nature of physiology in tetralogy of Fallot, specifically the contribution of infundibular narrowing during a “tet spell,” is not accurately represented by conventional 2D drawings. We hypothesize that the use of 3D models will improve residents’ knowledge acquisition and their overall satisfaction during the teaching session.

## Methods

With institutional review board approval and written informed consent, a prospective, randomized trial was conducted to compare the effectiveness of instruction to second year pediatric residents (PL-2 s) regarding tetralogy of Fallot, using a standard 2D depiction of the defect compared to using a 3D model of the defect. All other aspects of the teaching session, including content, teaching slides and didactic methods, were identical. Pre-session and post-session questionnaires were used to assess knowledge acquisition, learner satisfaction and self-efficacy ratings.

### Study participants

PL-2 s rotating through a 4-week block inpatient Cardiology rotation were recruited in groups of 2–3 at Children’s National Health System. There were 13 blocks per year. Participants were primarily from the affiliated pediatric residency programs. The teaching session is part of the standard morning curriculum the residents receive during their rotation. Other cardiology team members covered the PL-2 s’ patients by answering pages and interacting with nurses as required. Half the total participants received teaching sessions using 3D models, while the other half receiving sessions with 2D images were designated as control. Before each session, the participants filled out a questionnaire relating to their background and previous clinical exposure to patients with tetralogy of Fallot.

#### Education sessions

During the education session, PL-2 s were led through a lecture discussing several aspects related to tetralogy of Fallot – embryology, anatomical features, physiology, natural history, surgical approach and medical management of patients undergoing a cyanotic “Tet spell”. All teaching sessions were conducted by one of 2 co-investigators (ASH and LJO) with a standardized presentation. Each session was approximately 1 h in length. For the control group, 2D drawings were used during the session.

#### Educational session with physical 3D models

**Fig. 1:**
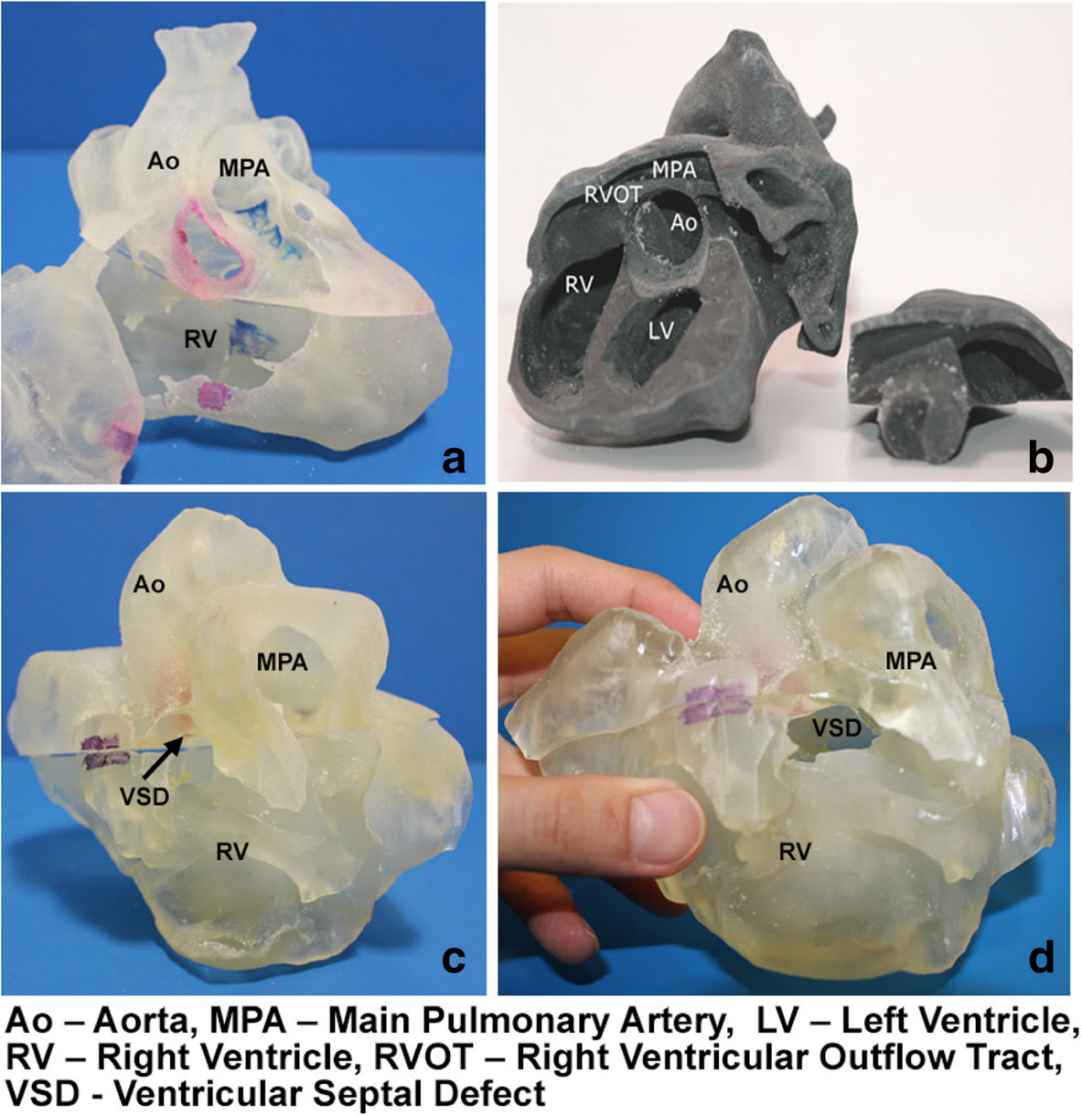
Respective physical models and features are as shown. **a** Normal heart: This model, created from Cardiac CT, is partitioned into 3 pieces, including an anterior portion (the right ventricular free wall) that can be removed to visualize the normal interventricular septum. The remaining superior and inferior portions can be separated to allow for visualization of the aorta and its position relative to the right ventricle. **b** Repaired tetralogy of Fallot heart from an adult: The model, created from Cardiac MRI is separated into 2 pieces, well fitted together via “Lego” peg depression. The cut in the main body allows for clear visualization of the pulmonary infundibular stenosis and overriding aorta. **c** Unrepaired tetralogy of Fallot heart from an infant: The 3D model, created from 3D echocardiogram, was partitioned into 2 pieces; a superior and inferior portion divided along the ventricular septal defect. **d** Unrepaired tetralogy of Fallot heart from an infant: Separating superior and inferior portions allows for clear visualization of the VSD as well as the aortic override relative to the VSD

In the intervention group, three physical 3D models were used in place of 2D images. The fabrication of physical models from cardiac imaging datasets with 3D segmentation software and a 3D printer has previously been described [10]. Software segmentation with commercial software (Mimics, Materialize, Belgium) was performed to create 3D digital models. The process of segmentation involves drawing contours between the endocardium and blood pool across a 3D volumetric dataset to form a 3D reconstruction. The models were fabricated from three separate cardiac imaging data sets that were anonymized: a cardiac CT of a normal infant heart, a cardiac MRI of an adult patient with repaired tetralogy of Fallot, and a 3D echocardiogram of an infant with unrepaired tetralogy of Fallot (Fig. 1). The models were divided and partitioned into several components for clear visualization of the ventricular septal defect (Fig. 1). The residents were allowed to hold the models, and were encouraged to take apart the models in order to point out key anatomical features such as the anteriorly malaligned ventricular septal defect.

### Outcome measures

#### Learner satisfaction and self-efficacy— (Kirkpatrick level One [11])

**Fig. 2:**
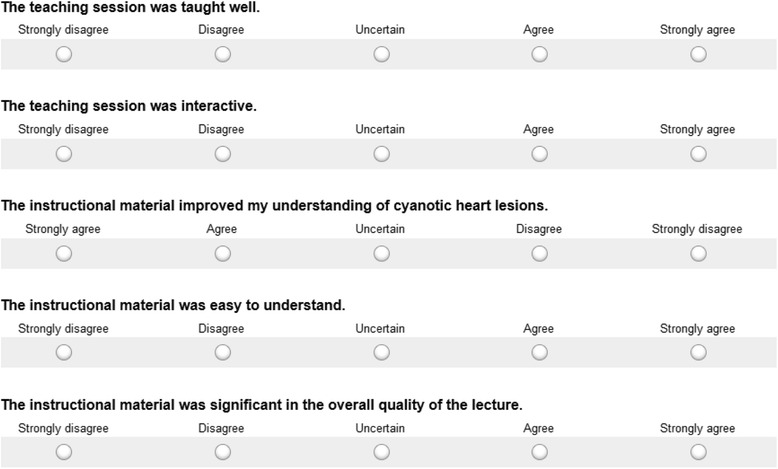
Questionnaire on learner satisfaction

**Fig. 3:**
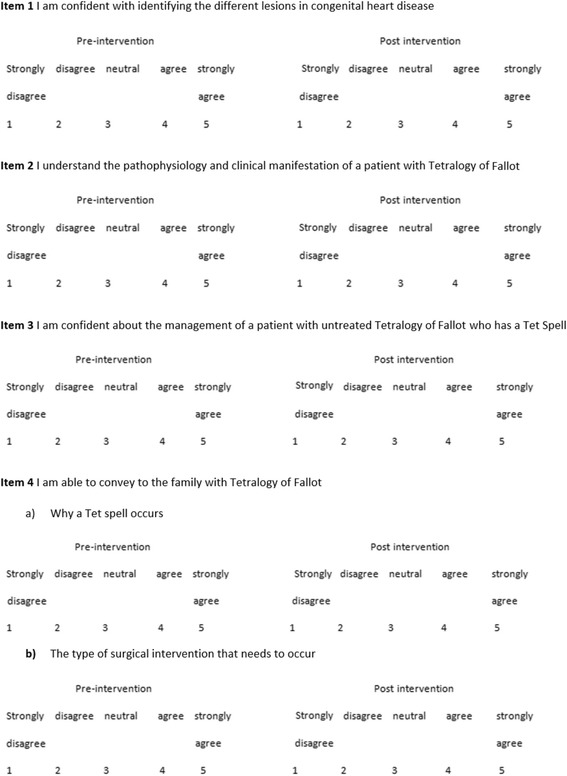
Questionnaire on self-efficacy

After the session, two questionnaires, each with 5 questions, were provided. The first questionnaire was designed to measure learners’ satisfaction with the teaching session (Fig. 2). The second questionnaire was designed to measure learners’ self-reported confidence in their knowledge of tetralogy of Fallot and its management (Fig. 3). Each question had a Likert scale rating from 1 to 5. For both questionnaires, composite score was measured with total possible scores ranging from 5 to 25.

#### Knowledge acquisition— (Kirkpatrick level Two [11])

Before the session, the participants were first given a pre-session knowledge test (in multiple choice question format, comprised of 9 questions). The questions tested the residents’ knowledge of the anatomy, physiology of tetralogy of Fallot, as well as the acute management of a patient with a “Tet spell”. After the teaching session, the participants were given the same knowledge test post-session. Correctly scored items were given a value of 1 and incorrect given values of 0. Pre-test and post-test scores were reported based on cumulative points (ranging from 0 to 9).

#### Statistical analysis

Comparisons between the test scores, learner satisfaction and self-efficacy questionnaires for the two groups were assessed. All data analyses were performed using statistical software (MedCalc 12.2.1.0, MedCalc Software, Bruges, Belgium). Knowledge acquisition was measured by the improvement in pre-test and post-test multiple choice questions. Paired *t*-test was used to compare pre-test and post-test score differences. *T*-test was also used to compare differences in learner satisfaction and self-efficacy between the two groups. Cronbach-alpha scores were conducted to assess for internal consistency, with results of 0.90 and 0.86 respectively.

## Results

Thirty-five pediatric PL-2 s were enrolled into the study. Seventeen residents were taught with 2D images, and eighteen residents were taught with 3D models. Participants were either affiliated with the residency program in the hospital or from a nearby pediatric residency program. Most residents had limited exposure (less than 4) to patients with tetralogy of Fallot prior to the lecture. The residents’ previous sources of knowledge were more commonly derived from lectures than clinical rounds (Table 1).

**Table 1:** Thirty-five pediatric residents enrolled into the study, with no significant differences in background characteristics, including previous clinical exposure to Tetralogy of Fallot

Education intervention	2D images	3D models	*P*-value
Number Enrolled	17	18	
Residency Track			0.7
Categorical	14	15	
Non-categorical	3	3	
Previous Exposure to # of ToF patients			0.6
None	7	8	
1–3 patients	9	6	
4–6 patients	1	2	
Previous sources of knowledge for ToF			0.9
Lectures	15	12	
Rounds	7	4	

**Fig. 4:**
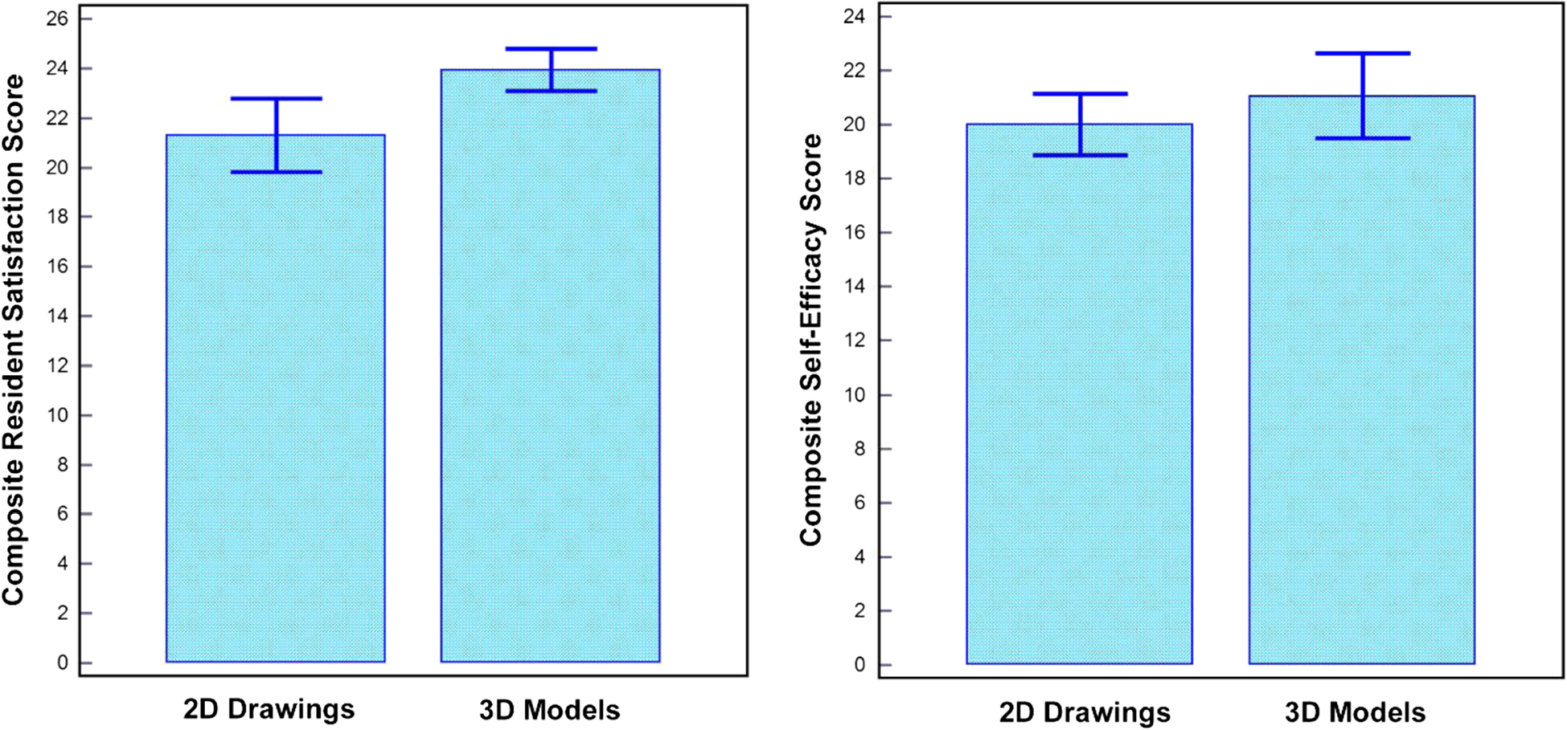
Impact of 3D heart models on medical education. Both 2D image and physical 3D model groups show improvement in knowledge based on multiple choice testing. There was a statistically significant difference in satisfaction ratings when 3D models were used. While residents in 3D model groups had higher self-efficacy aggregate scores, this difference was not statistically significant

Use of 3D models was found to improve the teaching sessions in several aspects (Fig. 4). First, residents reported better satisfaction when using 3D models compared to 2D images, with an improvement in composite learner satisfaction scores from 21 (84%) to 24 (96%) (*P* = 0.03). Second, the residents reported more confidence in their ability to manage patients with tetralogy of Fallot with a higher composite self-efficacy score, from 20 (80%) to 21 (84%). This difference was not statistically significant (*P* = 0.39).

Pediatric residents in both groups demonstrated similar knowledge-acquisition. Before the education sessions, pediatric residents in both groups demonstrated similar levels of knowledge based on pre-test score results. Following completion of the education sessions, pediatric residents had mean post-test scores of 6.3 (70%) and 6.0 (66%) respectively (Table 2). There was no statistical difference found in knowledge acquisition when using 2D images compared to 3D models. There was also no statistical difference found in relative improvement of test scores between 2D and 3D models.

**Table 2:** Impact of 3D heart models on knowledge acquisition. The pre-test and post-test comprised of 9 questions in multiple choice format. Both 2D image and 3D model groups show the same effect of knowledge acquisition

Study group	Mean pre-test score	Mean post-test score	*P*-value
2D Images	3.8 ± 1.5	6.3 ± 1.2	<0.01
3D Models	4.3 ± 1.9	6.0 ± 1.6	<0.01

## Discussion

Given the complexity of CHD, physical models are useful in demonstrating the relative locations of anatomic structures of interest, particularly with learners as demonstrated in this study. 3D models allow for easy manipulation and clear visualization of such structures. Anatomical and physiologic concepts of congenital heart disease can be demonstrated in a hands-on, interactive environment. Depth and spatial relationships can be expressed and elaborated. Printed 3D models have been previously used as an adjunct for cardiovascular surgeons in providing useful information such as spatial localization of coronary arteries [12], pre-operative planning for ventricular septal defects, and intra-operative orientation of complex vascular rings [8]. 3D models have also been helpful in guiding stent placements in complex CHD patients [13]. In tetralogy of Fallot, the use of a 3D model helps to specifically elaborate on the anterior deviation of the infundibular septum and how the shape of the right ventricular outflow tract can create different clinical presentations (whether a patient is predisposed to having an increased likelihood of a “tet spell”).

To our knowledge, this study is the first attempt to quantify the impact of 3D models in medical education of CHD in comparison to a control intervention. The use of 3D heart models has been previously applied in small curriculums for premedical, medical students and residents [14]. Interactive 3D models of the heart have also been used in education of transesophageal echocardiography [15]. Our study incorporated a control comparison with conventional 2D images. We found that 3D models helped improve residents’ satisfaction during the teaching sessions. The feedback forms also showed that residents found the models to be useful and engaging, similarly to Biglinio et al’s study. Being able to hold the heart and visualize the ventricular septal defect from different angles allowed residents to appreciate the size and location of the defect relative to the great vessels.

For learners in the clinical setting, increased satisfaction ratings are linked to increased long-term knowledge gains. A meta-analysis from Yammine et al. demonstrated that 3D physical models can improve long-term knowledge outcomes in understanding spatial arrangements of anatomic organs [16]. Previous educational studies have suggested that interactive educational techniques would help with facilitating resident learning as well as long-term retention of material [17–19]. Allowing hands-on learning with physical models inherently increases the interactivity of a session, potentially increasing learner’s knowledge retention of CHD. While long-term knowledge retention was not specifically assessed in this study, learner satisfaction was significantly different in the 3D model group and is known to correlate with retention in adult learners in other fields. Long-term knowledge retention will be formally assessed in future studies.

We did not find improvement in factual knowledge acquisition when using 3D models, a finding also demonstrated in Yammine et al’s meta-analysis. While the two groups didn’t have a significant difference in knowledge-based test scores, this is probably an appropriate outcome, as the learners are responsible for the material presented regardless of presentation with 2D or 3D teaching aids. Interestingly, Yammine’s meta-analysis distinguishes factual knowledge vs. spatial knowledge, which was not captured well with the knowledge acquisition testing. This is further compounded by the confounding factor of trainee level. Pediatric residents have limited exposure to patient with tetralogy of Fallot. Their previous knowledge of tetralogy of Fallot is mainly derived from lectures in medical school, where the physiology of tetralogy of Fallot is elaborated, but less so the spatial significance of an anterior malalignment ventricular septal defect. Understanding the complexities and nuances behind the adage “anatomy defines physiology” is still a roadblock, as reflected by the lower-than-expected post-test scores of both groups. This equipoise in knowledge acquisition was similarly demonstrated in Biglinio et al., which also did not show improvement in short term understanding of the child’s condition [7]. The potential benefit in knowledge acquisition may be better seen in medical trainees within the field of Pediatric Cardiology (pediatric cardiology fellows, critical care nurses and nurse practitioners) who utilize relevant spatial knowledge in the day-by-day management of congenital heart disease.

Creation of the 3D heart models via software segmentation can be effectively performed by a clinician familiar with both cardiac imaging and 3D software. For 3D echocardiogram datasets, low-gain acquisitions and post-processing filtering to remove speckling noise is required [20]. Additionally, 3D reconstructions should be optimized by manually segmenting respective atria, ventricles and great vessels as separate masks (with clear definition of the ventricular septal defect between masks) [10] and reconstructing the heart via Boolean function. Typically, software segmentation takes approximately 2 h for each model. Fabrication of physical models, however, remains quite resource and time-dependent. The models used in this study are fabricated with Objet500 Connex3 (Stratasys, Minnesota, USA), a multi-material 3D printer that utilizes PolyJet photopolymer materials. Materials with a rubbery consistency (Tango Black and Tango Clear) were used with an average cost of 1 US Dollar per gram of material. The overall cost for each model was approximately $200, although depending on the printer type this cost can range from about $15 to $300. The time to print each model was approximately 12 h. As a means of improving the cost-effectiveness of using 3D models for education, use of conventional digital display technologies (for example, a tablet with a 3D model viewer app that has the ability to manipulate, rotate and zoom in on a digital 3D heart) could dramatically reduce this cost.

There are several limitations to the study. This was a single center study with limited number of participants. As previously mentioned, the study was limited to teaching sessions involving pediatric residents only. Further studies should involve trainees in Cardiology, where there may be potential benefit in knowledge acquisition when learning more complex CHD. Knowledge acquisition was only reflected by a small number of multiple-choice questions. Follow-up testing was not performed in study participants to assess for knowledge retention or effect on patient care (Kirkpatrick Level Four) [11]. Assessment of learner satisfaction and self-efficacy were based on subjective Likert-scale ratings and not objective evaluation measures.

Future studies should examine the impact of models on teaching CHD that are more complex and elaborate, such as hypoplastic left heart syndrome. This critical CHD requires multiple surgeries with anatomic variation after each surgery, such that learners are faced with learning four different anatomies instead of just one [10]. Feedback forms will include questions related to the “most favorite model” in order to investigate the effectiveness in 3D models’ ability to communicate spatial information (and relevant anatomical details in CHD). Future work will also include impact of 3D models on education of male and female learners, given the published differences in spatial reasoning abilities between these two groups [21]. In light of the limitations of encountered in this study, more nuanced testing of trainee knowledge base should be used (such as incorporating free text answers or detailed use of the classroom critical incident questionnaire) [22] to fully assess understanding of spatial knowledge and specific anatomical variations.

## Conclusion

Our study shows that physical 3D models enhance resident education around the topic of congenital heart disease, specifically with tetralogy of Fallot, by improving learner satisfaction.

## Abbreviations

2D: Two-dimensional
3D: Three-dimensional
CHD: Congenital heart disease
